# Case Report: Frontalis sign for early bedside consideration of impending uncal herniation

**DOI:** 10.12688/f1000research.7871.2

**Published:** 2016-08-04

**Authors:** Sunil Munakomi, Bijoy Mohan Kumar

**Affiliations:** 1 Department of Neurosurgery, College of Medical Sciences, Bharatpur, Nepal

**Keywords:** Trauma, herniation, sign

## Abstract

It is prudent to have early diagnosis and timely management of uncal herniation for better management of neurosurgical patients. There are several clinical and radiological armamentariums that aid in early recognition of the condition. Through this case report, we try to highlight a simple bedside clinical sign that can be a valuable adjunct in early recognition of the impending uncal herniation especially in scenarios wherein it is difficult to assess the pupillary size and reactivity correctly. The improvement in the sign also confirms the resolution of the mass effect in the postoperative period. This is especially helpful for doctors working in the periphery or in resource restrained areas, for a timely referral of the patient to tertiary centre.

## Introduction

Traumatic brain injury (TBI) is now a global epidemic ^1^. The prognosis of patients with head injury is dependent on many clinical parameters but one of the major determinants is the time lapsed for appropriate management ^2^. TBI has a significant impact not only on the patients and their relatives but also has a major influence on the health and socioeconomical status in the global arena. Thus, it is prudent to have clinical tools for early recognition of life threatening neurosurgical emergencies. Herein we discuss one such example: Frontalis sign for detecting early uncal herniation. This may be helpful for early referral of patients to tertiary care centres and for timely management of the same. It could therefore have a positive impact on these patients, resulting in a better outcome.

## Case report

**Figure 1.**
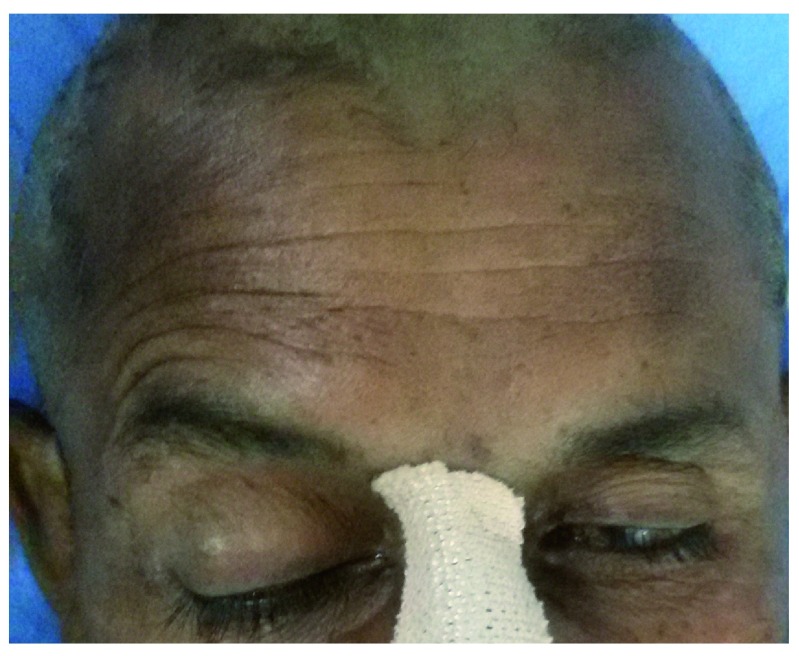
Prominence of wrinkles on the right forehead during attempted eye opening, along with ptosis termed as frontalis sign.

**Figure 2.**
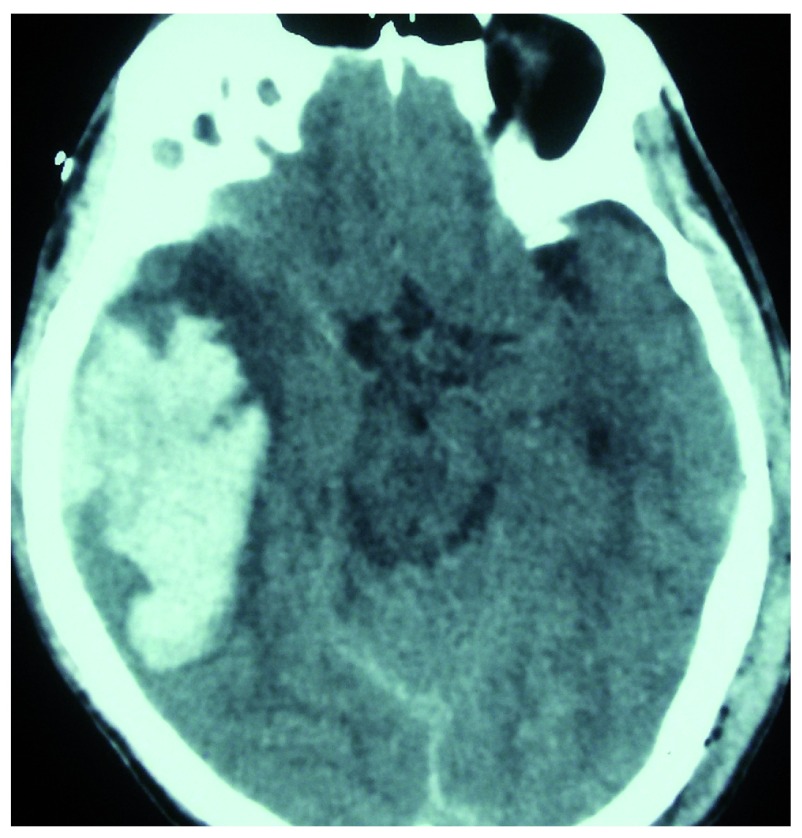
CT head image showing evidence of right sided uncal herniation with obliteration of the ipsilateral crural and the ambient cisterns following temporo-parietal huge contusion.

**Figure 3.**
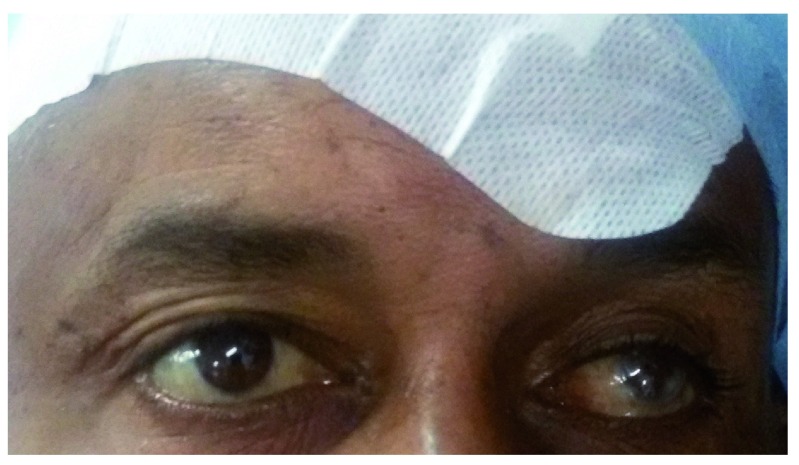
Resolution of the frontalis sign in the early postoperative period.

**Figure 4.**
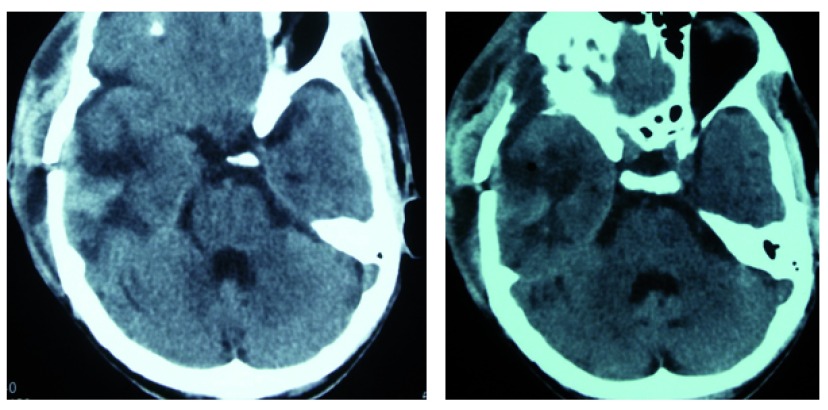
Postoperative CT scan confirmed resolution of the mass effect and normalization of the cisternal anatomy.

A 50-year-old male from Siraha, a distant village in Nepal, was referred to our neurosurgical centre following a road traffic accident after being hit by a speeding car. The patient had a brief loss of consciousness and a single episode of vomiting following the incident. There was no history of seizurogenic activity observed during the transfer. On arrival to the emergency department, his Glasgow coma scale (GCS) was E3M6V5 with no paucity in movement of any limbs. His vital parameters were within normal range with blood pressure of 130/90, pulse rate of 86/min and oxygen saturation of 99% in room air. It was difficult to assess differences in pupillary size as he had corneal opacity on the left eye, resulting from an injury sustained during his childhood. However, on close examination, we observed that there was prominence of the forehead wrinkles on the right half of his face especially when the patient was trying to open his eyes during conversation, which we termed as frontalis sign ( Figure 1). The wrinkles on the contra lateral half were normal with no abnormal deviation of angle of the mouth dismissing the differential diagnosis of upper facial nerve palsy. Because of the finding, we suspected impending uncal herniation in the patient and thereby advised for an emergency computed tomography (CT) scan of the head. It revealed right sided huge temporo-parietal contusion with thin fronto-temporo-parietal subdural hematoma with features of uncal herniation ( Figure 2). The condition was explained to his relatives and they were counseled for emergency evacuation of the hematoma. On their consent, we performed a craniotomy, evacuation of the subdural hematoma and removal of the contusion. Following the procedure, the brain was lax and pulsatile. The patient was extubated without any untoward events in the postoperative period. The frontalis sign diminished following the surgery ( Figure 3). The post operative scan confirmed resolution of the mass effect and normalization of the cisternal anatomy ( Figure 4). The patient was started on Levtiracetam 500 mg intravenously every 12 hours which was changed to oral medication after three days as seizure prophylaxis. The patient was discharged after suture removal on the 8th postoperative day. The patient followed up in the outpatient clinic 2 weeks later in sound health. Eye opening was near normal. The patient was advised for monthly follow up.

## Discussion

Levator palpebrae superioris supplied by the third nerve helps in elevation of the lid during eye opening ^4, 5^. However third nerve involvement due to uncal herniation weakens the muscle thereby restricting its action ^6^. In order to compensate the deficits, the frontalis belly of the occipito-frontalis muscle supplied by the facial nerve, helps in elevation of the lid ^7, 8^. This leads to prominence of forehead wrinkles in the same side on comparison to the other half. This is termed frontalis sign. This can act as a reliable bedside marker for diagnosing impending herniation. There are other routine signs for impending uncal herniation such as anisocoria. But there may be inter-observer bias in assessing the same ^9^. There may be other causes for anisocoria such as Horner’s syndrome following carotid artery dissection, traumatic third nerve palsy, Marcus gun pupil (Relative afferent pupillary defect) and ocular perforations ^10^. Sometimes drugs such as Ipratropium Bromide used for nebulisation in the intensive care unit can cause anisocoria. It is also difficult to observe the size and reaction of the pupils in patients with severe eye lid swellings ^11^. The frontalis sign can be used as an adjunct for consideration of uncal herniation and thereby initiating the correct management. This is even more valuable for proper patient referral from peripheral and resource limited setups, especially in developing countries like ours who are still far behind implementing the guidelines for managing patients with TBI ^12^.

## Conclusion

The implications of the use of this simple bedside sign for early diagnosis of the uncal diagnosis can be influential in providing timely and correct therapeutic targets for patients with TBI. It can be a valuable adjunct to the present panoply of our armamentarium in diagnosis the traumatic cerebral herniation syndromes.

## Consent

Written informed consent was obtained from the daughter of the patient for publication of this case report and any accompanying images and/or other details that could potentially reveal the patient’s identity.
