# Supplementary material for: Case Report: Frontalis sign for early bedside consideration of impending uncal herniation
Source: F1000Res. 2016 Aug 4;5:125. Originally published 2016 Feb 1. [Version 2] doi: 10.12688/f1000research.7871.2 (PMC5017293; doi:10.12688/f1000research.7871.2)
Supplement: Peer review history

# Peer review: Referee response for version 2

**DOI:** 10.5256/f1000research.10092.r13404

**Published:** 2016-09-05

**Authors:** G. Bryan Young

**Affiliations:** 1 Department of Clinical Neurological Sciences, University of Western Ontario, London, ON, Canada

The revision is well done and any queries have been addressed.

I have read this submission. I believe that I have an appropriate level of expertise to confirm that it is of an acceptable scientific standard.

# Peer review: Referee response for version 1

**DOI:** 10.5256/f1000research.8472.r15318

**Published:** 2016-07-29

**Authors:** Osman Sinanovic, Sanela Zukić

**Affiliations:** 1 Department of Neurology, Acta Medica Saliniana, University Clinical Center of Tuzla, Tuzla, Bosnia and Herzegovina; 2 Department of Neurology, University Clinical Center Tuzla, Tuzla, Bosnia and Herzegovina

In this case report authors try to highlight a simple bedside clinical sign (frontalis sign) in early recognition of the impending uncul herniation.

                                          

 Title “Frontalis sign for early bedside diagnosis of impending uncal herniation" is appropriate for content of the article but with a clinical sign we can not mkae adiagnosis. So, title “Frontalis sign for early bedside consideration of  impending uncal herniation” may be more appropriate.

 Article content including design, case report and discussion is correct.

We have read this submission. We believe that we have an appropriate level of expertise to confirm that it is of an acceptable scientific standard.

# Peer review: Referee response for version 1

**DOI:** 10.5256/f1000research.8472.r13921

**Published:** 2016-05-20

**Authors:** G. Bryan Young

**Affiliations:** 1 Department of Clinical Neurological Sciences, University of Western Ontario, London, ON, Canada

The paper is of interest. The authors should give details of pupillary reactivity. With herniation there is usually a change in pupillary size and reactivity before the ptosis. Was this not the case here?

I have read this submission. I believe that I have an appropriate level of expertise to confirm that it is of an acceptable scientific standard, however I have significant reservations, as outlined above.

# Response

**Published:** 2016-07-03

**Authors:** Sunil Munakomi

We thank you for the report on our article.We acknowledge the role of assessing pupillary size in patients with trauma. Here, we are emphasizing on the adjunct role of frontalis sign in diagnosing early uncal herniation in cases wherein pupillary assessment is problematic as in severe eye lid swelling,corneal injuries (in our case), following use of sedatives or following post traumatic seizure.
